# Curcumin alleviates heatstroke-induced liver injury in dry-heat environments by inhibiting the expression of NF-κB, iNOS, and ICAM-1 in rats

**DOI:** 10.1371/journal.pone.0309598

**Published:** 2024-09-06

**Authors:** Xinyue Yang, Liang Xia, Caifu Shen, Jiajia Li, Xiang Dong, Jiangwei Liu

**Affiliations:** 1 Key Laboratory of Special Environmental Medicine of Xinjiang, Urumqi, China; 2 Graduate School of Xinjiang Medical University, Urumqi, China; 3 Beijing Luhe Hospital Affiliated to Capital Medical University, Beijing, China; ABEx Bio-Research Center, BANGLADESH

## Abstract

we aimed to monitor liver injury in rat model during heat stress and heatstroke in dry-heat environment and investigate the effects of curcumin on heatstroke-induced liver injury and the underlying mechanisms. Sprague-Dawley (SD) rats were randomly divided into four groups: normal saline (NS), and 50 (50-cur), 100 (100-cur), and 200 mg/kg curcumin (200-cur) groups. They were administered the indicated doses of curcumin by gavage once daily for 7 days. On day 8, the rats were transferred to a simulated climate cabin, At 0, 50, 100, and 150 min, the core temperature (Tc) was measured respectively. After sacrificing the rats, tissue samples were collected, measure histology indices, serum enzymes, lipopolysaccharides (LPSs), cytokines, nuclear factor-kappa B (NF-κB), inducible nitric oxide synthase (iNOS), and intercellular adhesion molecule-1 (ICAM-1). The Tc increased with time in all groups. Curcumin alleviation of symptoms and improvement in pathological scores. The level of enzymes, LPS, and cytokines increased during heatstroke in the NS group, but curcumin decreased the levels of these indicators. The differences of the indicators between NS and 200-cur groups at 150 min were significant (P < 0.05). The expression of NF-κB p65, iNOS, and ICAM-1 was upregulated in the NS group at 150 min, but their expression was relatively lower in the curcumin groups (P < 0.05). Thus, our findings indicate acute liver injury during heat stress and heatstroke. The mechanism involves cascade-amplification inflammatory response induced by the gut endotoxin. Furthermore, curcumin alleviated heatstroke-induced liver injury in a dose-dependent manner by downregulating NF-κB, iNOS, and ICAM-1.

## Introduction

Heatstroke is a potentially fatal event that usually occurs at high temperatures during the summer. Heatstroke in a dry-heat environment is characterized by high body temperature, minimal perspiration, and encephalopathy [1]. It does not readily occur but can cause irreversible internal organ injury and may even be fatal. Individuals who are exposed to dry-heat environments for extended periods, such as workers, soldiers, travelers, the sick, and the elderly individuals, are comparatively more susceptible to heatstroke. The dry-heat environment is characteristic of gobies and deserts, and these are usually located in remote areas. Consequently, patients may not be quickly transferred to medical facilities or treated efficiently. Heatstroke prevention and treatment in dry-heat environments are essential, but the availability of drugs and other measures in these environments is limited.

Heatstroke is defined as "a form of hyperthermia associated with a systemic inflammatory response leading to a syndrome of multiple organ dysfunction in which encephalopathy predominates" [2]. Current research suggests that the systemic inflammatory response is induced by an endotoxin from the intestinal tissue injured during heat stress. The endotoxin enters the liver via the portal vein, injures the liver, and causes multiple organ dysfunction syndrome [3].

Liver function and injury are involved in the occurrence and development of heatstroke [4]. However, the mechanism of heatstroke-induced liver injury is controversial. Based on studies in human patients and animal models of heatstroke, it has been proposed that the gut endotoxin activates Kupffer cells, which form the central link of an inflammatory cascade [3,5,6]. Nuclear factor-kappa B (NF-κB) regulates the expression of cytokines and proinflammatory mediators [7] such as inducible nitric oxide synthase (iNOS) and intercellular adhesion molecule-1 (ICAM-1). These are usually expressed in macrophages and endothelial cells during inflammation. They may play important roles in the pathophysiology of secondary liver injury induced by heatstroke.

Curcumin is extracted from the rhizome of Curcuma longa (Zingiberaceae), which is used in Traditional Chinese Medicine. It has been widely used in the treatment of several diseases. Its anti-oxidant, anti-inflammatory, and anticancer properties have been investigated [8–10]. Studies have shown that curcumin attenuates liver injury induced by drugs, poisons, and intestinal ischemia-reperfusion by inhibiting NF-κB expression [11–13]. In contrast, information on the hepatoprotective effects of curcumin during heatstroke is limited.

To the best of our knowledge, the application of curcumin in the treatment of heatstroke has not been reported. In the present study, we investigated whether curcumin protects rat liver against injury during heatstroke in a dry-heat environment. We also tried to elucidate the protective mechanism by measuring the expression levels of hepatic NF-κB, iNOS, and ICAM-1 in response to curcumin administration.

## Materials and methods

### Animals

Male Sprague–Dawley rats, each weighing 190–220 g, were purchased from Animal Resource Center of Xinjiang Medical University of China (license number SCXK (Xin) 2018–0002). The study protocol was approved by the Institutional Ethics Committee of the General Hospital of Xinjiang Military Region. Animal feeding and care were in accordance with the National Science Council guidelines.

### Experimental design

The rats were adaptively fed for 7 days in a specific pathogen-free room at 20°C ± 2°C and 40%–50% relative humidity (RH), under a 12-h light-dark cycle. All 160 rats were randomly divided into four groups of 40 each: normal saline (NS), 50 mg/kg curcumin (50-cur), 100 mg/kg curcumin (100-cur), and 200 mg/kg curcumin (200-cur) groups. The NS group received a normal saline gavage and the 50-cur, 100-cur, and 200-cur groups received curcumin by gavage at 1 mL 100 g-1 doses daily for 7 days. Curcumin (Cl-753000, Nippon Kasei Co. Ltd., Japan) was prepared as a suspension using 0.5% w/v carboxymethylcellulose sodium (CMC-Na). All rats had ad libitum access to sterile food and water.

On day 8, 30 rats per group were transferred to a simulated climate cabin for special environments in Northwest China, Urumqi, China, under the following conditions: 41°C ± 0.5°C, 10% ± 1% RH, and restricted food and water intake. Based on a previous study [1], at 50, 100, and 150 min after the start of the study, the core temperature (Tc) of 10 rats per group was measured using a polygraph system (BL420F; Chengdu TME Technology Co. Ltd., Chengdu, China). The other 10 rats per group in the specific pathogen-free room were moved outside, and their Tc was measured and recorded as the Tc at 0 min. The rats were anesthetized by 5% pentobarbital sodium injection into the abdominal cavity (30 mg/kg), then extract venous blood from the inferior vena cava and collect liver tissues. The blood samples were divided into several blood tubes, centrifuged (1500 × g, 10 min, 25°C), and stored at -20°C. The liver tissues were divided into two pieces. One was fixed with 10% neutral buffered formalin and the other was stored in liquid nitrogen (-196°C) for the subsequent analyses. All animal sampling operations were performed under intraperitoneal anesthesia and after the elimination of corneal and pain reflexes, under sterile conditions, and died painlessly due to blood exhaustion under continuous anesthesia. All animal procedures are strictly in accordance with international ethical standards and the guidelines for the care and use of experimental animals issued by the National Institutes of Health. They have been approved by the Animal Ethics Committee of the Xinjiang Military Region General Hospital and comply with the 3R principle. The approval number is DWLL20201009.

### Liver histology

The liver tissues were collected, fixed with 10% neutral buffered formalin, embedded in paraffin, and stained with hematoxylin and eosin. The films were read by animal histopathology professionals under double-blind conditions under double-blind conditions. Ten random visual fields were randomly examined for each histopathological section under a microscope at 10×10 magnification, and the average histopathological injury scores were calculated. Based on heatstroke-induced liver damage symptoms [14,15], a liver injury scoring system was established as follows: hepatocyte degeneration and necrosis (3), edema (1), or none (0); central veins in the lobules in liver thrombosis (3), congestion (1), or none (0); hepatic sinusoid dilation and congestion (1) or none (0); inflammatory cell infiltration in the hepatic sinusoid rated serious (3), moderate (2), mild (1), or none (0). Each histopathological section was evaluated and the liver injury scores were calculated.

### Measurement of serum alanine transaminase (ALT) and aspartate transaminase (AST)

The blood samples were placed in blood tubes with heparin and separating gel and centrifuged (1500 × g, 10 min, 25°C) to obtain serum samples. The ALT and AST levels in the serum were measured using an automatic biochemical analyzer (Mindray BS-180; Mindray Medical Electronics Co. Ltd., Shenzhen, China).

### Measurement of plasma lipopolysaccharides (LPSs)

The blood samples were placed in blood tubes with heparin and separating gel and centrifuged (1500 × g, 10 min, 25°C) to obtain the supernatants. The LPS level in the supernatant was measured using a tachypleus reagent kit (Chinese Horseshoe Crab Reagent Manufactory, Xiamen, China) according to the manufacturer’s instructions.

### Measurement of hepatic cytokines

Approximately 0.1 g of liver tissue was ground in liquid nitrogen and diluted with 0.9 mL of 1× phosphate-buffered saline (PBS). The suspension was centrifuged (13,800 × g, 3 min, 4°C) and the supernatant obtained was divided in four separate Eppendorf tubes. Protein concentrations were determined using a bicinchoninic acid (BCA) protein assay kit (Thermo Fisher Scientific, Waltham, MA, USA). The level of tumor necrosis factor-alpha (TNF-α), interleukin (IL)-1β, IL-6, and IL-10 was measured using enzyme-linked immunosorbent assay (ELISA; Joyee Biotechnics, Hefei, China) kits according to the manufacturer’s instructions, and the data were analyzed together with those of the BCA protein assay.

### Western blotting

Approximately 0.2 g of liver tissue was removed from the liquid nitrogen container and ground with liquid nitrogen. The total protein was extracted with 2 mL of protein lysis buffer (50 mM Tris-HCl (pH 8.0), 150 mM NaCl, and 0.1% w/v sodium dodecyl sulfate (SDS)) containing 1% NP-40, 0.1 mM ethylenediaminetetraacetic acid (EDTA), 1×protease inhibitor cocktail, and 1 mM phenylmethylsulfonyl fluoride. The liver tissues were lysed on ice water for 1 h at 4°C with intermittent rotation every 10 min. After centrifugation (13,800 × *g*, 3 min, 4°C), the homogenates were mixed with 2× sodium dodecyl sulfate-polyacrylamide gel electrophoresis (SDS-PAGE) loading buffer. The total protein extract was separated by 8%–10% SDS-PAGE (The loading amount of protein for SDS-PAGE was 48μg), transferred onto polyvinylidene fluoride membranes, blocked with bovine serum albumin, and incubated with the primary antibodies anti-ICAM-1 (1:500, ab171123; Abcam, Cambridge, UK) and iNOS (1:500, ab3523; Abcam) and the HRP-conjugated secondary anti-mouse antibodies (1:3000, ab97023; Abcam) and anti-rabbit antibodies (1:3000, ab97051; Abcam). β-Actin was used as the control (1:2000, A1978; Sigma-Aldrich Corp., St. Louis, MO, USA).

Another 0.2 g of liver tissue was ground and added to 2 mL of lysis buffer A (10 mM HEPES (pH 7.9), 10 mM KCl, 0.1 mM EDTA, 0.1 mM ethylene glycol-bis(β-aminoethyl ether)-N,N,N′,N′-tetraacetic acid (EGTA), 0.5% NP-40, 2 mM dithiothreitol (DTT), and 1× EDTA-free protease inhibitor). After rotating for 10 min at 4°C and centrifuging (13,800 × *g*, 3 min, 4°C), the supernatant was discarded, and the sediment volume was estimated. To the sediment, 3× volume of lysis buffer B (20 mM HEPES (pH 7.9), 0.4 M NaCl, 1 mM EDTA, 1 mM EGTA, 1 mM DTT, and 1× EDTA-free protease inhibitor) were added. After incubating for 30 min at 4°C with strong intermittent vortexing followed by centrifuging (13,800 × *g*, 3 min, 4°C), the final supernatant consisting of nucleoprotein extracts was collected and used for the detection of NF-κB p65 (1:500, ab7970; Abcam). The analytical method was the same as that used to detect the tissue proteins. Histone-H2A (1:1,000, ab45152; Abcam) was used as the control. The data were processed using a gel-imaging analysis system (UVP 510 Imager; Thermo Fisher Scientific).

### Statistical analysis

The data are expressed as mean ± SD. One-way ANOVA and a rank-sum test were conducted to identify differences among groups. All statistical analyses were performed using SPSS v. 21 (IBM Corp., Armonk, NY, USA). Differences were considered statistically significant at *P* < 0.05.

## Results

### Curcumin delayed body temperature increase during heat stress and heatstroke in a dry-heat environment

**Fig 1:**
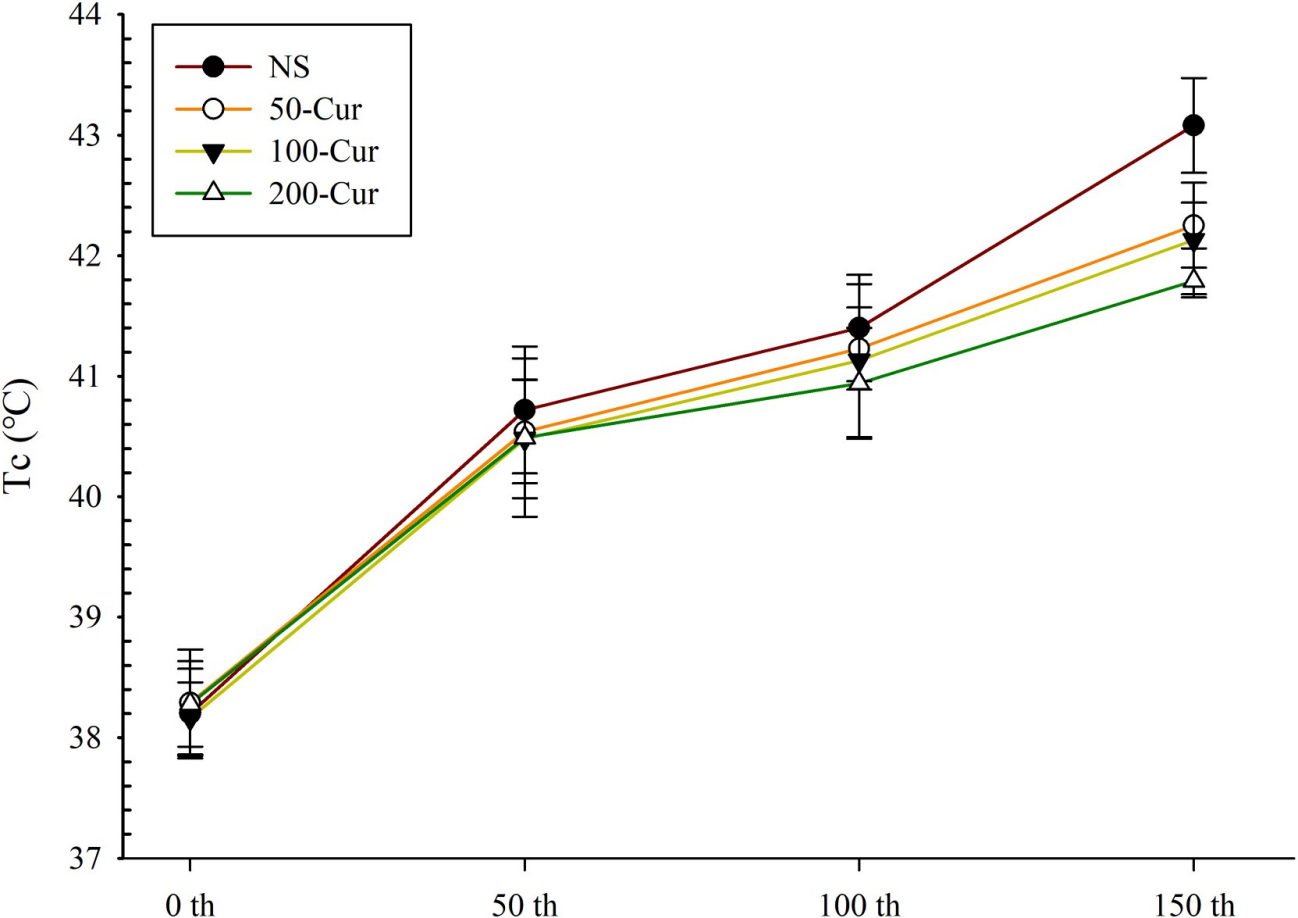
Effect of curcumin on the core temperature (Tc) during heat stress and heatstroke in rats. n = 10/group. NS, normal saline, 50-cur, 50 mg/kg curcumin, 100-cur, 100 mg/kg curcumin, and 200-cur, 200 mg/kg curcumin groups.

As shown in Fig 1 and Table 1, with the prolongation of time, the Tc of all four groups gradually increased. The Tc of the four groups increased rapidly in the first 50 min, slowly in the following 50 min, and further accelerated in the last 50 min. The Tc of the NS group at 50, 100, and 150 min was significantly higher than that at 0 min (*P*<0.05). The trend of Tc change is consistent with our previous research [1]. Tc sharply increases during heat stress, reaches a plateau, and then rapidly increases. There was no significant difference in Tc between the groups at 0 and 50 min. At 100 min, the Tc of the 200-cur group was significantly lower than that of the NS group. At 150 min, the Tc of the 50-cur, 100-cur, and 200-cur groups were significantly lower than those of the NS group (*P*<0.05), and the Tc of the four groups showed that the NS group>50-cur>100-cur>200-cur group, indicating a concentration dependent effect of curcumin.

**Table 1:** Effect of curcumin on the Tc (°C) during heat stress and heatstroke in rats.

Group	0 min	50 min	100 min	150 min
NS	38.20 ± 0.37	40.72 ± 0.53^#^	41.40 ± 0.44^#^	43.08 ± 0.40^#^
50-cur	38.29 ± 0.44	40.54 ± 0.43	41.23 ± 0.34	42.25 ± 0.19^*^
100-cur	38.16 ± 0.30	40.48 ± 0.49	41.13 ± 0.63	42.13 ± 0.48^*^
200-cur	38.28 ± 0.36	40.49 ± 0.66	40.94 ± 0.46^*^	41.79 ± 0.11^*^

Data are expressed as mean ± SD.

^#^ *P* < 0.05, compared with the NS group at 0 min

^*^ *P* < 0.05, compared with the NS group.

### Curcumin improved liver histopathology during heatstroke in a dry-heat environment

**Fig 2:**
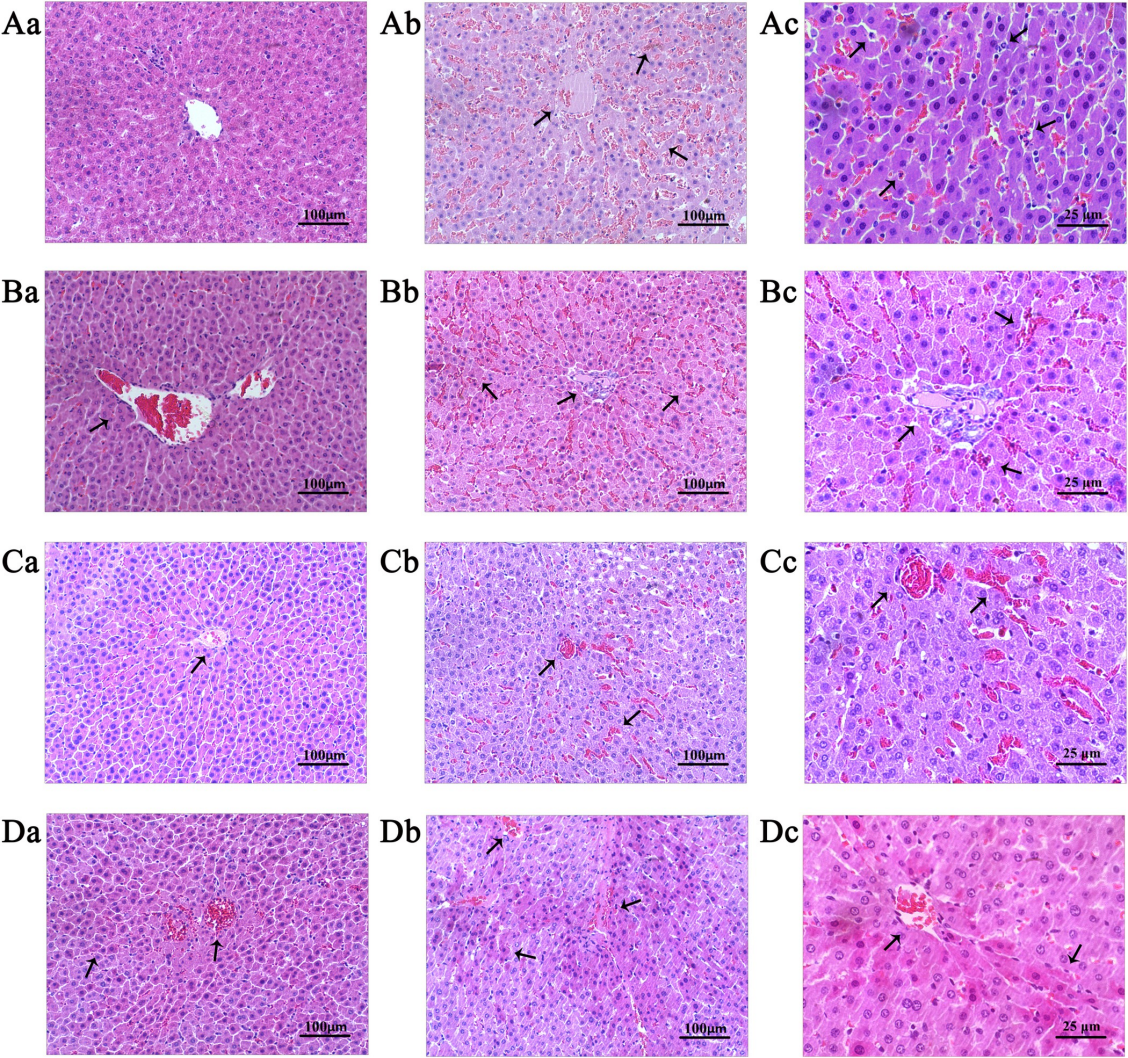
Effects of curcumin on rat liver pathology during heatstroke. n = 10/group. NS, normal saline, 50-cur, 50 mg/kg curcumin, 100-cur, 100 mg/kg curcumin, and 200-cur, 200 mg/kg curcumin groups. Scale bar = 100 μm. Aa. NS 0^th^ group; Ab, Ac. NS 150^th^ group; Ba. 50-cur 0^th^ group; Bb, Bc. 50-cur 150^th^ group; Ca. 100-cur 0^th^ group; Cb, Cc. 100-cur 150^th^ group; Da. 200-cur 0^th^ group; Db, Dc. 200-cur 150^th^ group.

**Fig 3:**
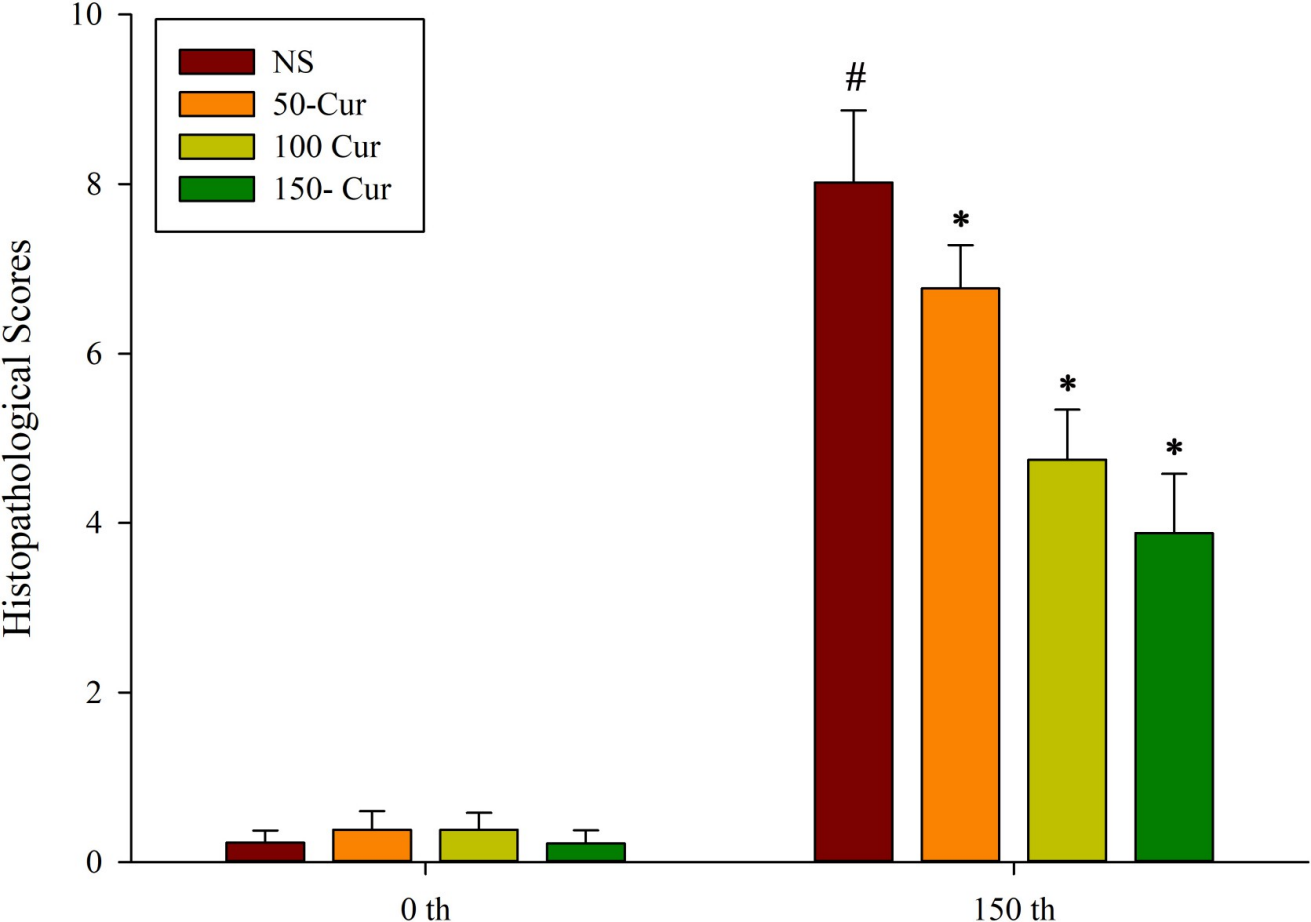
**Effects of curcumin on rat liver-histopathology scores during heatstroke**. n = 10/group. All data are presented as the mean ± SD. One-way ANOVA with LSD’s post-hoc test was performed. NS, normal saline, 50-cur, 50 mg/kg curcumin, 100-cur, 100 mg/kg curcumin, and 200-cur, 200 mg/kg curcumin groups. ^#^ *P* < 0.05, compared with the NS group at 0 min; ^*^ *P* < 0.05, compared with the NS group.

As shown in Fig 2, at 0 min, there were no significant histopathological changes (Aa, Ba, Ca, Da) in the liver of the four groups of rats. At 150 min, the histopathological changes in the liver of NS group rats were mainly manifested as hepatocyte edema, eosinophilic changes, central venous thrombosis, hepatic sinus dilation and congestion, and neutrophil infiltration (Ab, Ac). Compared with the NS group, the pathological changes in the 50-cur, 100-cur, and 200-cur groups were reduced (Bb, Bc, Cb, Cc, Db, Dc), especially in the 200-cur group, liver cell edema was reduced, central venous dilation and congestion, degree of thrombosis, and infiltration of inflammatory cells were reduced. As shown in Fig 3, there was no significant difference in liver histopathological injury scores among the four groups at 0 min. The liver injury score in the NS group significantly increased at 150 min and was higher than at 0 min (*P*<0.05). At 150 min, the liver injury scores of the 50-cur, 100-cur, and 200-cur groups were significantly lower than those of the NS group (*P*<0.05); And the NS group>50-cur group>100-cur group>200-cur group, suggests that the hepatoprotective effect of curcumin may be concentration dependent.

### Curcumin decreased the ALT and AST levels during heat stress and heatstroke in a dry-heat environment

**Fig 4:**
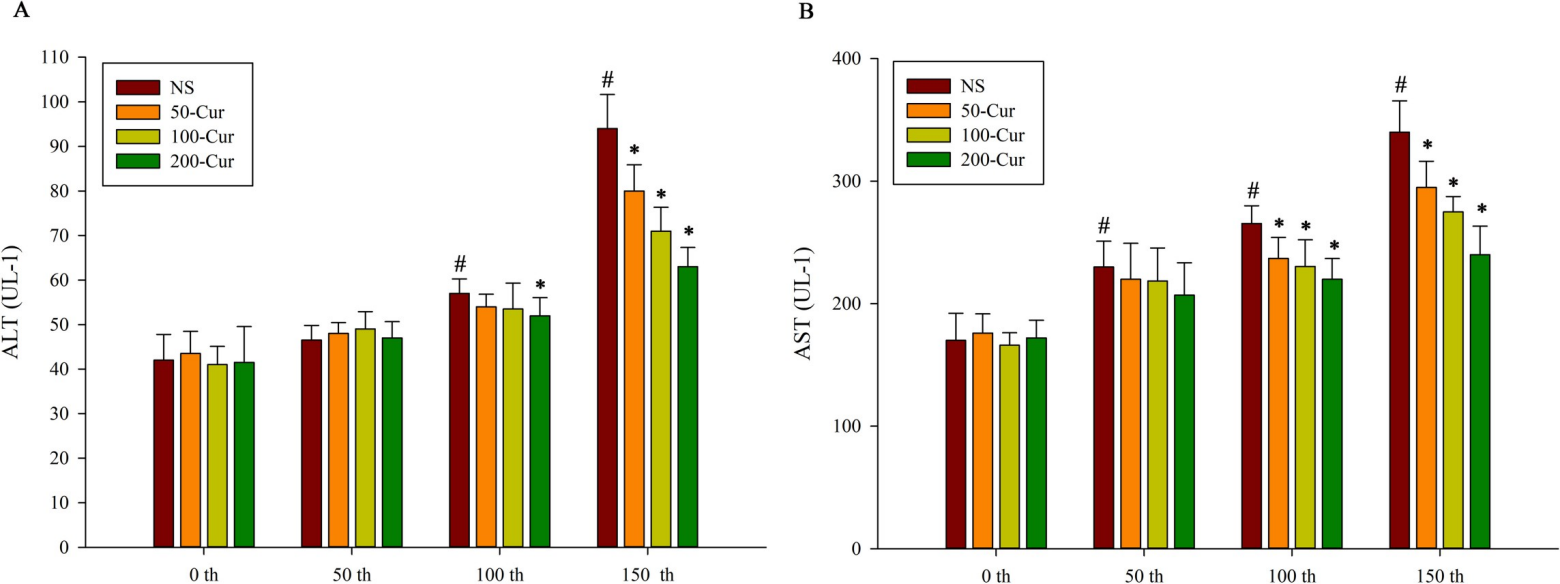
Effects of curcumin on serum enzymes during heat stress and heatstroke in rats. A, alanine transaminase (ALT), B, aspartate transaminase (AST), n = 10/group. All data are presented as the mean ± SD. One-way ANOVA with LSD’s post-hoc test was performed. NS, normal saline, 50-cur, 50 mg/kg curcumin, 100-cur, 100 mg/kg curcumin, and 200-cur, 200 mg/kg curcumin groups. ^#^ *P* < 0.05, compared with the NS group at 0 min; ^*^ *P* < 0.05, compared with the NS group.

As shown in Fig 4A and 4B, the serum levels of ALT and AST in the four groups of rats showed a gradually increasing trend with prolonged exposure time. Compared with 0 min, the ALT levels in the NS group significantly increased at both 100 and 150 min (*P*<0.05). At 0 and 50 min, there was no significant difference in ALT levels among the four groups. At 100 min, the ALT level in the 200-cur group significantly decreased and was lower than that in the NS group (*P*<0.05). At 150 min, the ALT levels of the four groups were presented as follows: NS>50-cur>100-cur>200-cur group. The ALT levels in the 50-cur, 100-cur, and 200-cur group decreased and were lower than those in the NS group (*P*<0.05). Compared with 0 min, the AST levels in the NS group significantly increased at 50, 100, and 150 min (*P*<0.05). At 0 and 50 min, there was no significant difference in AST levels among the four groups. At 100 and 150min, the AST levels of all four groups showed NS>50-cur>100-cur>200-cur. At these two time points, the ALT levels in the 50-cur, 100-cur, and 200-cur group all decreased and were lower than those in the NS group (*P*<0.05).

### Curcumin reduced the LPS level in circulation during heat stress and heatstroke in a dry-heat environment

**Fig 5:**
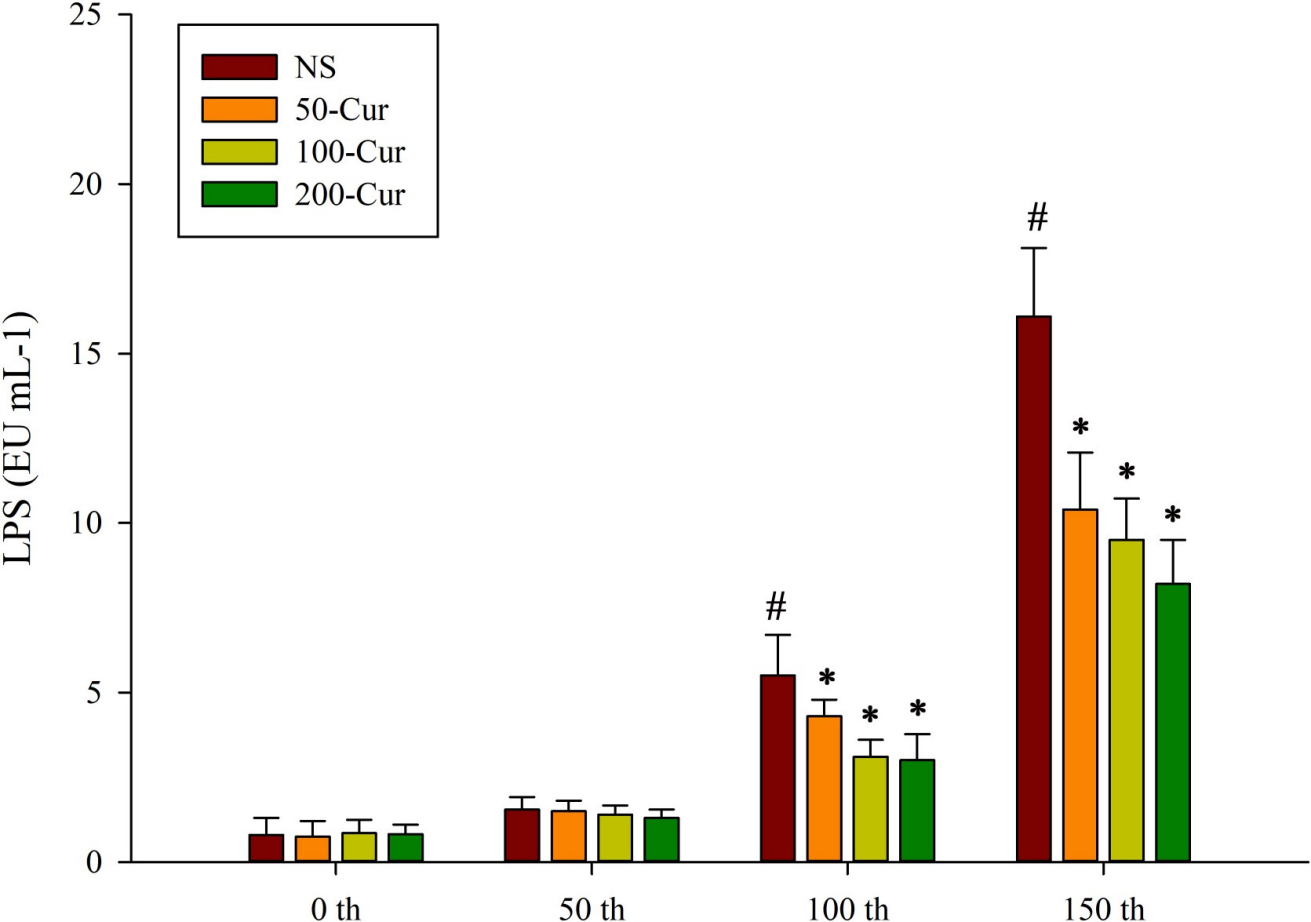
Effect of curcumin on LPS during heat stress and heatstroke in rats. Lipopolysaccharide (LPS). n = 10/group. All data are presented as the mean ± SD. One-way ANOVA with LSD’s post-hoc test was performed. NS, normal saline, 50-cur, 50 mg/kg curcumin, 100-cur, 100 mg/kg curcumin, and 200-cur, 200 mg/kg curcumin groups. ^#^ *P* < 0.05, compared with the NS group at 0 min; ^*^ *P* < 0.05, compared with the NS group.

As shown in Fig 5, the serum LPS levels of rats in the four groups showed a gradually increasing trend with the prolongation of exposure time. Compared with 0 min, the LPS level in the NS group significantly increased at both 100 and 150 min (*P*<0.05). At 0 and 50 min, there was no significant difference in LPS levels among the four groups. At 100 and 150 min, the LPS levels of all four groups showed NS>50-cur>100-cur>200-cur. The LPS levels in the 50-cur, 100-cur, and 200-cur group decreased and were lower than those in the NS group at these two time points (*P*<0.05).

### Curcumin reduced hepatic cytokine levels during heat stress and heatstroke in a dry-heat environment

**Fig 6:**
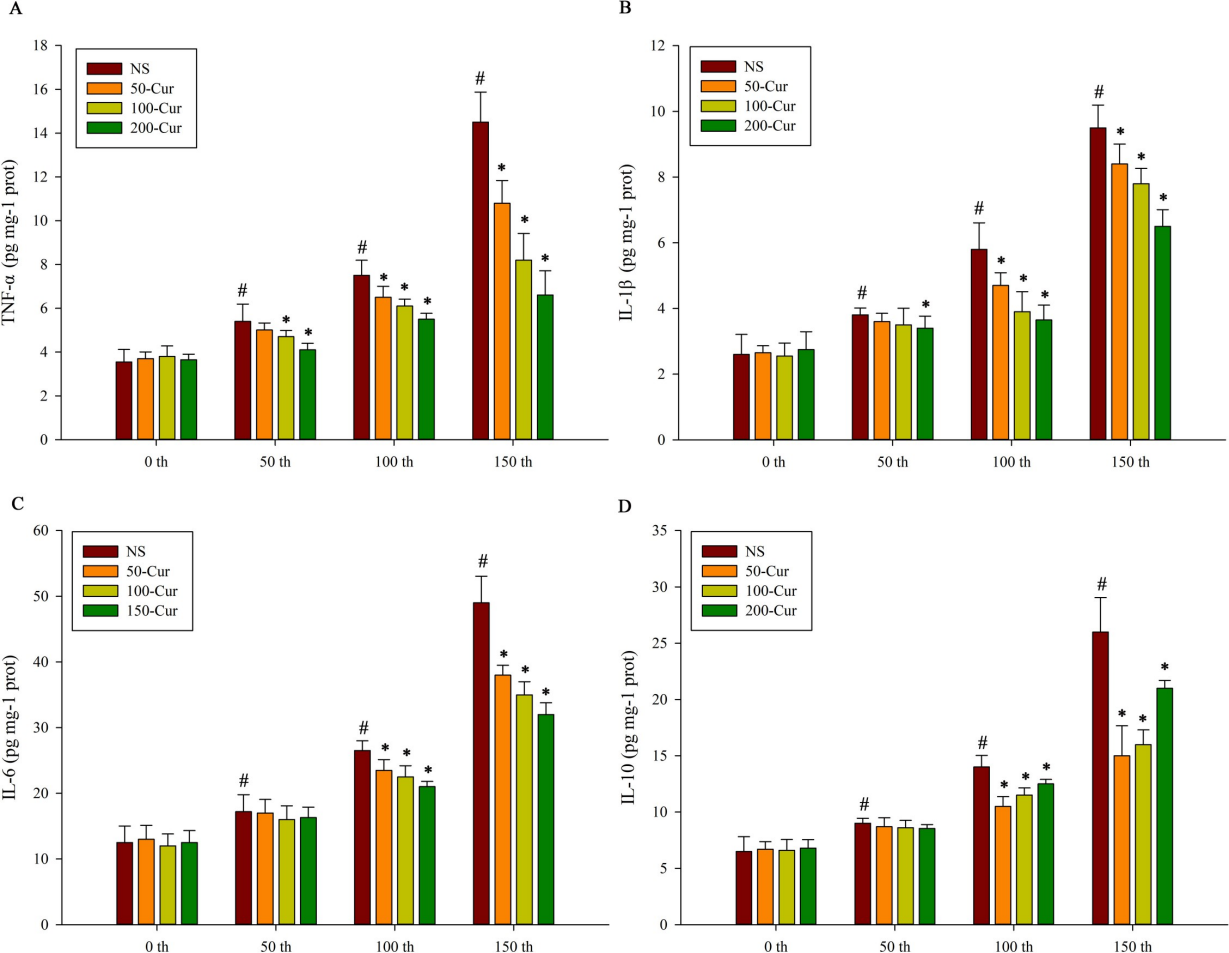
Effects of curcumin on hepatic cytokines during heat stress and heatstroke in rats. A, Tumor necrosis factor (TNF)-α, B, Interleukin (IL)-1β, C, IL-6, and D, IL-10. n = 10/group. All data are presented as the mean ± SD. One-way ANOVA with LSD’s post-hoc test was performed. NS, normal saline, 50-cur, 50 mg/kg curcumin, 100-cur, 100 mg/kg curcumin, and 200-cur, 200 mg/kg curcumin groups. ^#^ *P* < 0.05, compared with the NS group at 0 min; ^*^ *P* < 0.05, compared with the NS group.

As shown in Fig 6A–6D, the levels of TNF-α, IL-1β, IL-6, and IL-10 in the liver of rats from the four groups showed a gradually increasing trend with the prolongation of exposure time, indicating inflammatory liver injury. Compared with 0 min, the levels of TNF-α, IL-1β, IL-6, and IL-10 in the NS group significantly increased at 50, 100, and 150 min (*P*<0.05). At 0 min, there was no significant difference in the levels of TNF-α, IL-1β, IL-6, and IL-10 among the four groups. At 50 min, the TNF-α levels in both the 100-cur and the 200-cur group decreased and were lower than those in the NS group (*P*<0.05), while the IL-1β levels in the 200-cur group decreased and were lower than those in the NS group (*P*<0.05). At 100 and 150 min, the TNF-α, IL-1β, and IL-6 water of the four groups showed: NS >50-cur>100-cur>200-cur group. At these two time points, the TNF-α, IL-1β, and IL-6 levels in the 50-cur, 100-cur, and 200-cur group decreased on average and were lower than those in the NS group (*P*<0.05). At 100 and 150 min, compared with the NS group, the IL-10 levels in the 50-cur, 100-cur, and 200-cur group decreased and were lower than those in the NS group (*P*<0.05), and the 200-cur> 100-cur>50-cur group.

### Curcumin reduced the expression of NF-κB in the liver during heatstroke in a dry-heat environment

**Fig 7:**
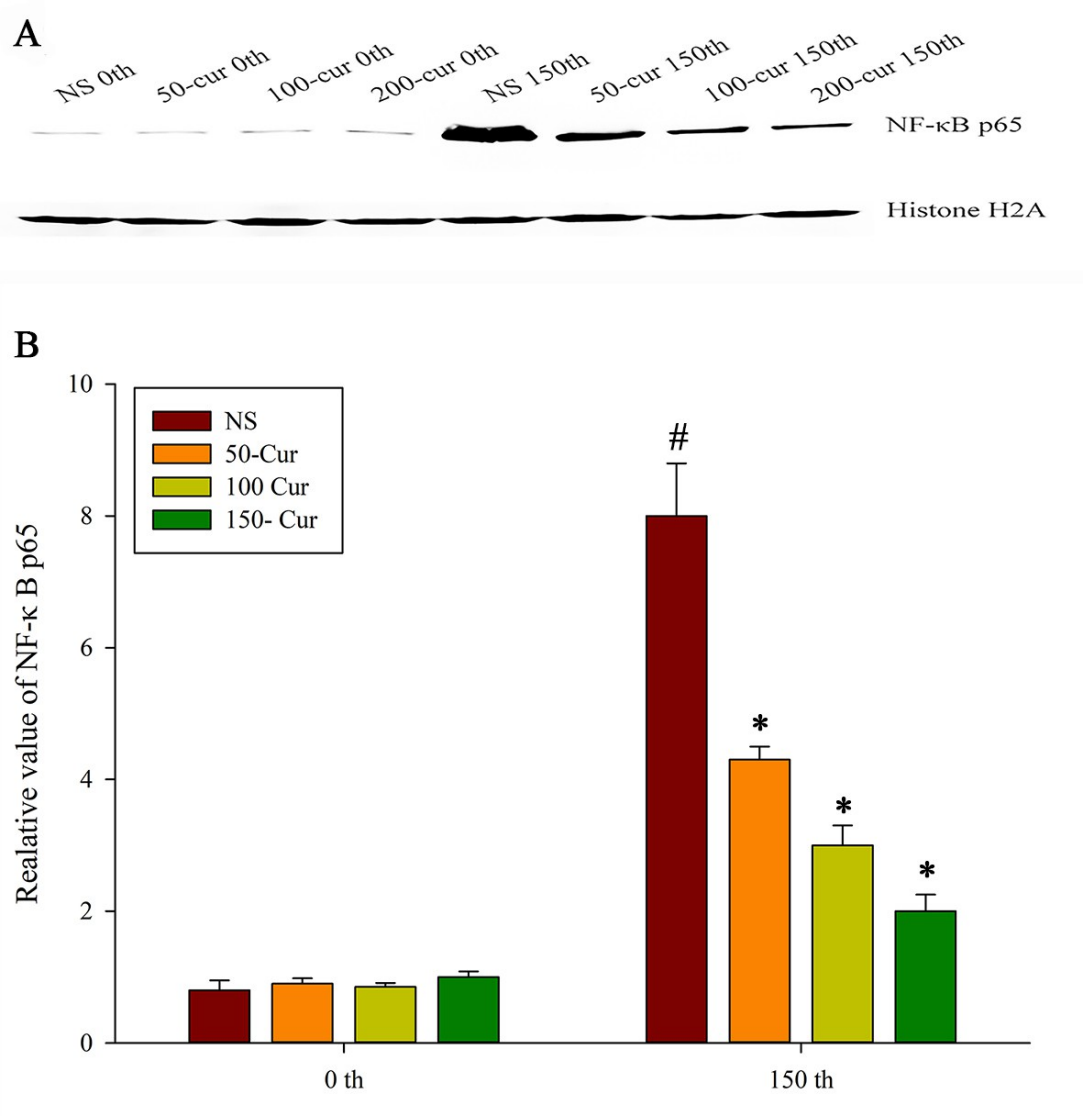
Effect of curcumin on hepatic NF-κB p65 during heatstroke in rats. A, Immunoblot results of NF-κB p65, B, relative value of NF-κB p65. n = 10/group. All data are presented as the mean ± SD. One-way ANOVA with LSD’s post-hoc test was performed. NS, normal saline, 50-cur, 50 mg/kg curcumin, 100-cur, 100 mg/kg curcumin, and 200-cur, 200 mg/kg curcumin groups. ^#^ *P* < 0.05, compared with the NS group at 0 min; ^*^ *P* < 0.05, compared with the NS group.

As shown in Fig 7A and 7B), the NF-κB p65 expression in the liver of the four groups of rats increased with prolonged exposure time. Compared with 0 mins, the NF-κB p65 expression in the NS group significantly increased at 150 min (*P*<0.05). At 0 min, there was no significant difference in NF-κB p65 expression among the four groups. At 150 min, the NF-κB p65 expression of the four groups showed NS>50-cur>100-cur>200-cur group. The NF-κB p65 expression in the 50-cur, 100-cur, and 200-cur group decreased and were lower than those in the NS group (*P*<0.05).

### Curcumin downregulated hepatic iNOS and ICAM-1 during heatstroke in a dry-heat environment

**Fig 8:**
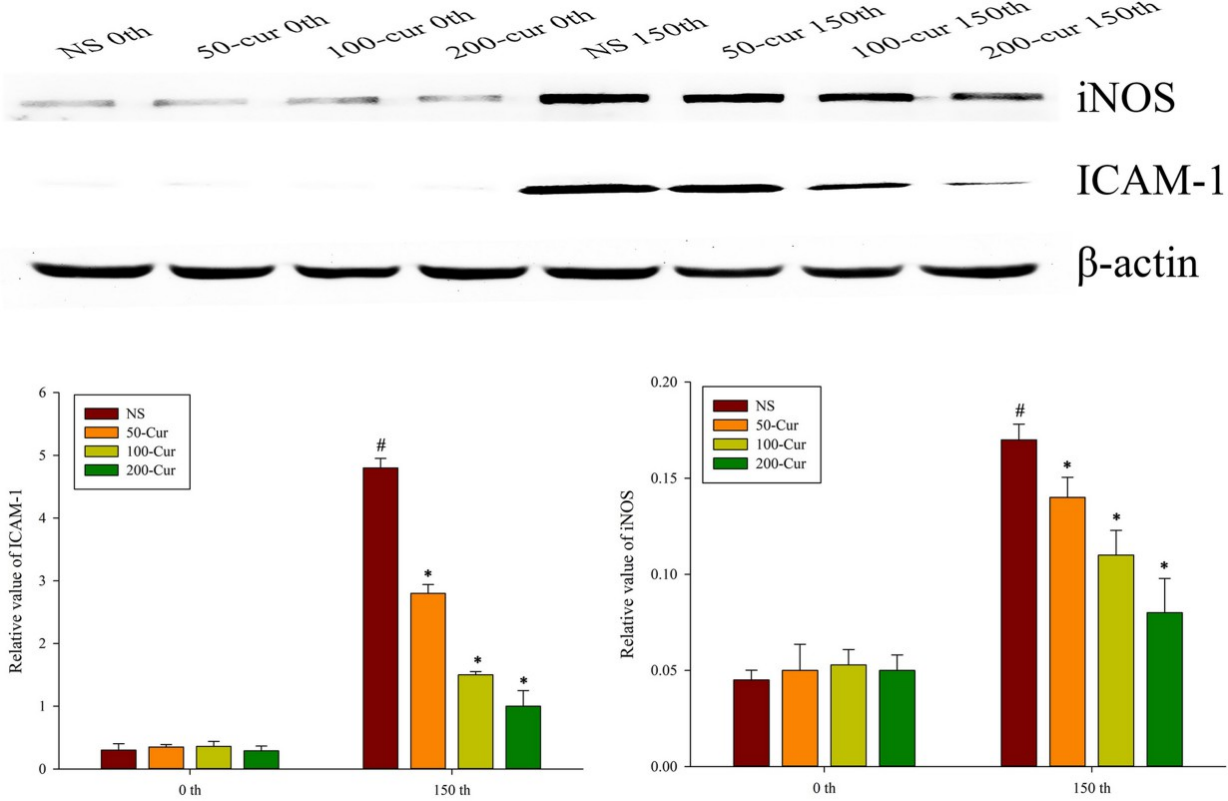
Effects of curcumin on hepatic iNOS and ICAM-1 expression during heatstroke in rats. A, Immunoblot results of ICAM-1 and iNOS, B, relative value of ICAM-1, C, relative value of iNOS. n = 10/group. All data are presented as the mean ± SD. One-way ANOVA with LSD’s post-hoc test was performed. NS, normal saline, 50-cur, 50 mg/kg curcumin, 100-cur, 100 mg/kg curcumin, and 200-cur, 200 mg/kg curcumin groups. ^#^ *P* < 0.05, compared with the NS group at 0 min; ^*^ *P* < 0.05, compared with the NS group.

As shown in Fig 8A–8C, the expression of iNOS and ICAM-1 in the liver of rats in the four groups increased with prolonged exposure time. Compared with 0 min, the expression of iNOS and ICAM-1 in the NS group significantly increased at 150 min (*P*<0.05). At 0 min, there was no significant difference in iNOS and ICAM-1 levels among the four groups. At 150 min, the iNOS and ICAM-1 levels in the four groups showed NS>50-cur>100-cur>200-cur group. The expression of iNOS and ICAM-1 in the 50-cur, 100-cur, and 200-cur group decreased and were lower than those in the NS group (*P*<0.05).

## Discussion

Heatstroke is a serious disease often leading to multiple organ dysfunction syndrome in its terminal stage. Secondary organ injury may be caused by direct heat, inflammation, and disseminated intravascular coagulation [16,17]. Despite fluid resuscitation and cooling measures, the temperature of patients with heatstroke continues to increase, and the clinical manifestation resembles that of sepsis [18,19]. The liver is readily affected by heatstroke, and the inflammatory response induced by the endotoxin plays an important role in liver injury.

In the present study, we monitored the Tc of rats during the three stages of progression from heat stress to heatstroke [20], namely, thermoregulation during the compensatory phase, an acute phase of response, and a decompensation phase. As the environment conditions of the present study were severe (41°C ± 0.5°C, 10% ± 1% RH), the Tc rapidly increased in the first stage. The amount of heat directly absorbed exceeded that dissipated from the body by radiation. This mechanism is more likely to occur in a dry-heat environment than in a humid-heat environment. The plateau stage indicates a relative balance between heat absorption and dissipation. In this stage, the production of stress proteins is initiated to protect against tissue injury and promote cell repair. A sharp increase in the Tc after the plateau indicates that the circulation has failed, compensatory thermoregulation response has collapsed, and break-repair balance has been disrupted. It is believed that the latter is caused by the endotoxin and inflammatory response induction. Nevertheless, curcumin retards the increase in the Tc and prolongs the plateau.

The liver is a lymphoid organ that forms a part of the immune system [21]. When the intestinal barrier is broken during heat stress [22,23], LPSs enter the liver via the portal vein and are phagocytized by Kupffer cells in the hepatic sinusoid. In this way, the cells are activated. Toll-like receptor 4 (TLR4) on the cell membrane recognizes LPSs and initiate the TLR4 signaling pathway, which activates and transfers NF-κB p50/p65 to the nucleus, and then pro- and anti-inflammatory cytokines are transcribed and released [24,25]. Constant LPS stimulation promotes cytokines such as IL-1β and TNF-α to combine with receptors on the cell membrane, activate NF-κB, and trigger the cascade amplification of an unregulated inflammatory response [26]. On the contrary, cytokines activate macrophages and endothelial cells and upregulate iNOS and ICAM-1 expression, which break the hepatic sinusoid barrier, recruit neutrophils, and enable LPSs to evade the immune system and either directly attack hepatocytes or enter circulation [27]. These effects may induce thrombosis, which blocks liver microcirculation and causes hepatocytic hypoxia. Together, these mechanisms result in liver injury.

Liver pathology and serum ALT and AST levels are indices of acute or chronic liver injury. Acute liver failure commonly occurs during heatstroke [28,29]. The ALT and AST levels are reliable markers of liver injury. Elevated ALT and AST levels (Fig 3), congestion, and inflammatory cell infiltration of the hepatic sinusoid (Fig 2) are signs of inflammatory liver injury during heatstroke. Nevertheless, curcumin administration reversed liver pathology and restored normal serum ALT and AST levels in a concentration-dependent manner.

Curcumin is extracted from the rhizome of *C*. *longa*, which has been widely used and extensively studied in medicine. It has been applied for its antioxidant and anti-inflammatory properties. In the present study, curcumin decreased the levels of cytokines and downregulated NF-κB p65, iNOS, and ICAM-1 expression during heatstroke, and thereby alleviated liver injury.

Cytokines may participate in the progression of heatstroke by mediating fever, recruiting leukocytes, and activating endothelial cells [30]. IL-1 knockout mice presented with minimal liver injury, faster recovery, and better prognosis than the wild type during heatstroke [31]. IL-1 β regulated liver apoptosis during heat stress and heatstroke [14]. IL-6 attenuated the release of proinflammatory cytokines, such as TNF-α, in response to endotoxin stimulation. This mechanism was verified in IL-6 knockout mice whose survival rate increased relative to the wild type during heatstroke [32]. In the present study, we observed that the changes in the cytokine (TNF-α, IL-1β, IL-6, and IL-10) levels in the liver were consistent with liver injury during heatstroke. Curcumin decreased both pro- and anti-inflammatory cytokine levels. There were highly similar between the change tendency of pro- and anti-inflammatory cytokine levels in the NS and curcumin groups. This indicates that both pro- and anti-inflammatory cytokines were simultaneously released during heatstroke in response to the acute inflammatory response induced by LPS. Moreover, the target of curcumin was upstream of the NF-κB signaling pathways.

NF-κB is a transcription factor expressed in the nucleus in response to inflammation. The NF-κB family includes Rel A (p65), Rel B, c-Rel, NF-κB1 (p50/p105), and NF-κB2 (p52/p100). In immune responses, NF-κB mediates an inflammatory response and regulates cytokine expression. NF-κB is activated by LPS, cytokines, growth factors, and other stimuli [33–35]. It has been suggested that curcumin protects the liver against the harmful effects of sepsis, ischemia-reperfusion, radiation and acute intoxication by inhibiting the NF-κB pathways [36–39]. In the present study, curcumin decreased intranuclear NF-κB p65 expression in the liver after 150 min of heatstroke. Thus, it may regulate cytokine expression. A decrease in the cytokine levels suppresses positive feedback from inflammation and protects the liver from injury.

Inflammatory signals often involve the promotion of iNOS, which produces nitric oxide (NO). NO may serve as a protective mechanism against ischemia/reperfusion-induced liver injury [40]. However, iNOS overexpression generates large quantities of NO and causes peroxynitrite-related damage [41]. The enzyme iNOS is present in both immune cells and non-immune endothelial cells [42]. It has been reported that iNOS expression of kidney was upregulated in a dry-heat environment [43]. A study showed that iNOS upregulation caused liver injury and sinusoidal endothelial dysfunction. In contrast, iNOS downregulation attenuated liver injury by reducing nitro-oxidative stress in endotoxemic rats [44]. Selective iNOS inhibition mitigated acute sepsis-induced organ injury and improved survival [45]. NF-κB p65 and p50 regulated iNOS expression in myeloid cells [46]. In the present study, the dry-heat environment induced an inflammatory response and upregulated iNOS expression in the liver, which could explain hepatic sinusoid and barrier breakage. Nevertheless, this mechanism was blocked by curcumin via the inhibition of NF-κB.

Cytokines caused endothelial cell injury in hepatic sinusoid. IL-1β activated endothelial cells and induced cytokine and ICAM-1 release [47]. As the endothelial cells in hepatic sinusoid filter the blood arriving from the portal vein, they serve as a barrier to circulation in the liver [48]. Once these cells are injured, reactive oxygen species and endotoxins from the gut enter circulation and trigger systemic inflammatory response syndrome. The gut bacteria induce liver injury by activating Kupffer cells and stimulating ICAM-1 release from sinusoidal endothelial cells [49]. ICAM-1 recruit inflammatory cells, block microcirculation, aggravate the oxygen debt accrued during heatstroke as a result of high metabolic demand, and exacerbate liver injury [50]. In the present study, we observed thrombi in the central and portal vein areas and neutrophil infiltration in the hepatic sinusoid of the NS group. In contrast, these characteristics were alleviated in the curcumin groups at 150 min (Fig 2). ICAM-1 expression was upregulated in the NS group compared with that in the curcumin groups (Fig 7). Therefore, curcumin may protect sinusoidal endothelial cells and preserve endothelial structure.

As shown in Fig 4, the level of LPSs was lower in the curcumin groups than in the NS group at 150 min. Curcumin may have inhibited NF-κB expression and protected hepatocytes and sinusoidal endothelial cells from inflammatory injury, thereby keeping the sinusoidal endothelium intact, maintaining efficient liver metabolism, and preventing LPS from entering circulation. Nevertheless, the level of LPSs in circulation was 0.8 ± 0.5 EU per mL at 0 min. This phenomenon could be explained by the increased reaction time range, which in turn increased all values.

To the best of our knowledge, the present study is the first to evaluate curcumin in the treatment of heatstroke. Our data indicate that its effects were positive and beneficial. Here, curcumin displayed an anti-inflammatory effect. It inhibited NF-κB and protected the liver against inflammatory injury during heatstroke. It also alleviated blood clotting and protected the hepatic sinus barrier by downregulating iNOS and ICAM-1 expression. Elucidation of the mechanism of liver injury during heatstroke and the potential targets and hepatoprotective effects of curcumin in heatstroke may have clinical importance. In future research, we will administer curcumin by injection to rats with heatstroke and continue to assess its therapeutic effects.

## Conclusions

In the present study, we demonstrated that acute liver injury occurs during heat stress and heatstroke in a dry-heat environment. This response was mediated by a cascade-amplification inflammatory response induced by the gut endotoxin. The inflammatory mediators iNOS and ICAM-1 may play important roles in liver injury. Curcumin may alleviate liver injury by inhibiting the expression of NF-κB, iNOS, and ICAM-1, indicating the hepatoprotective effect of curcumin during heatstroke in a dry-heat environment.

## Supporting information

S1 Fig (TIF)

S2 Fig (TIF)

S3 Fig (TIF)

S4 Fig (TIF)

S5 Fig (TIF)

S1 Data (XLSX)

S1 File (XLSX)
