# Supplementary figures and images for: Curcumin alleviates heatstroke-induced liver injury in dry-heat environments by inhibiting the expression of NF-κB, iNOS, and ICAM-1 in rats
Source: PLoS One. 2024 Sep 6;19(9):e0309598. doi: 10.1371/journal.pone.0309598 (PMC11379272; doi:10.1371/journal.pone.0309598)

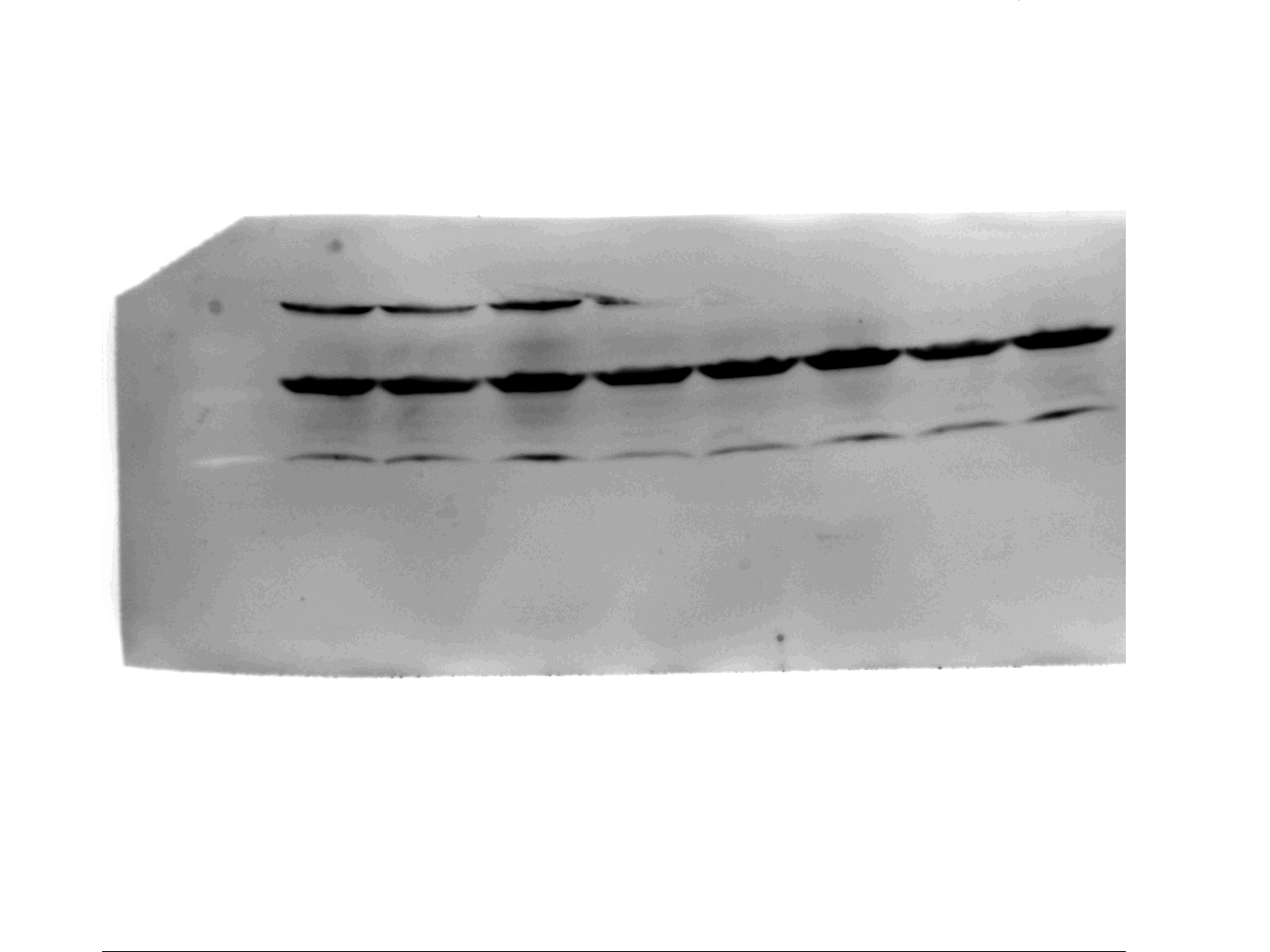

Supplement: S1 Fig — (TIF) [file pone.0309598.s001.TIF]

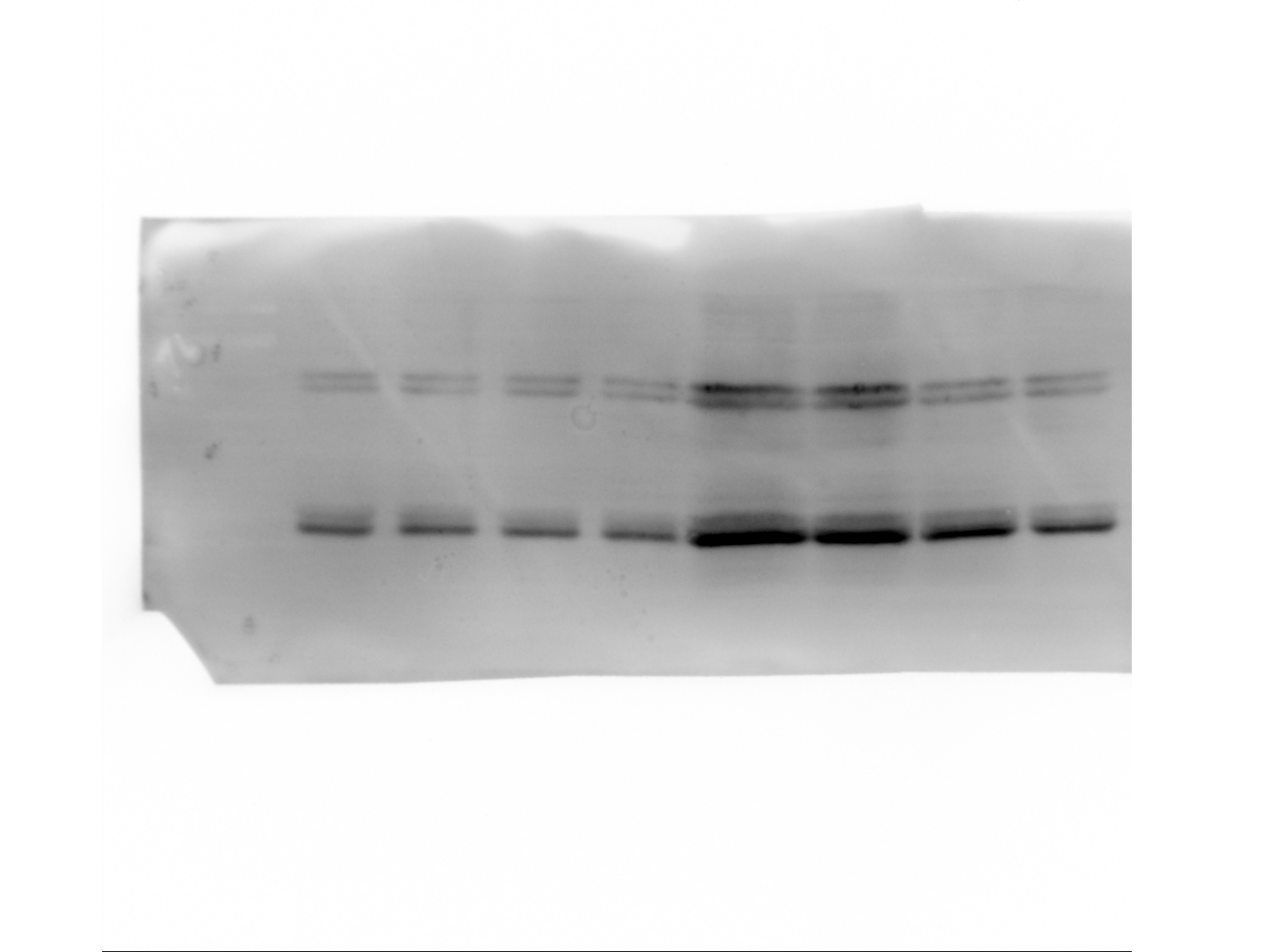

Supplement: S2 Fig — (TIF) [file pone.0309598.s002.TIF]

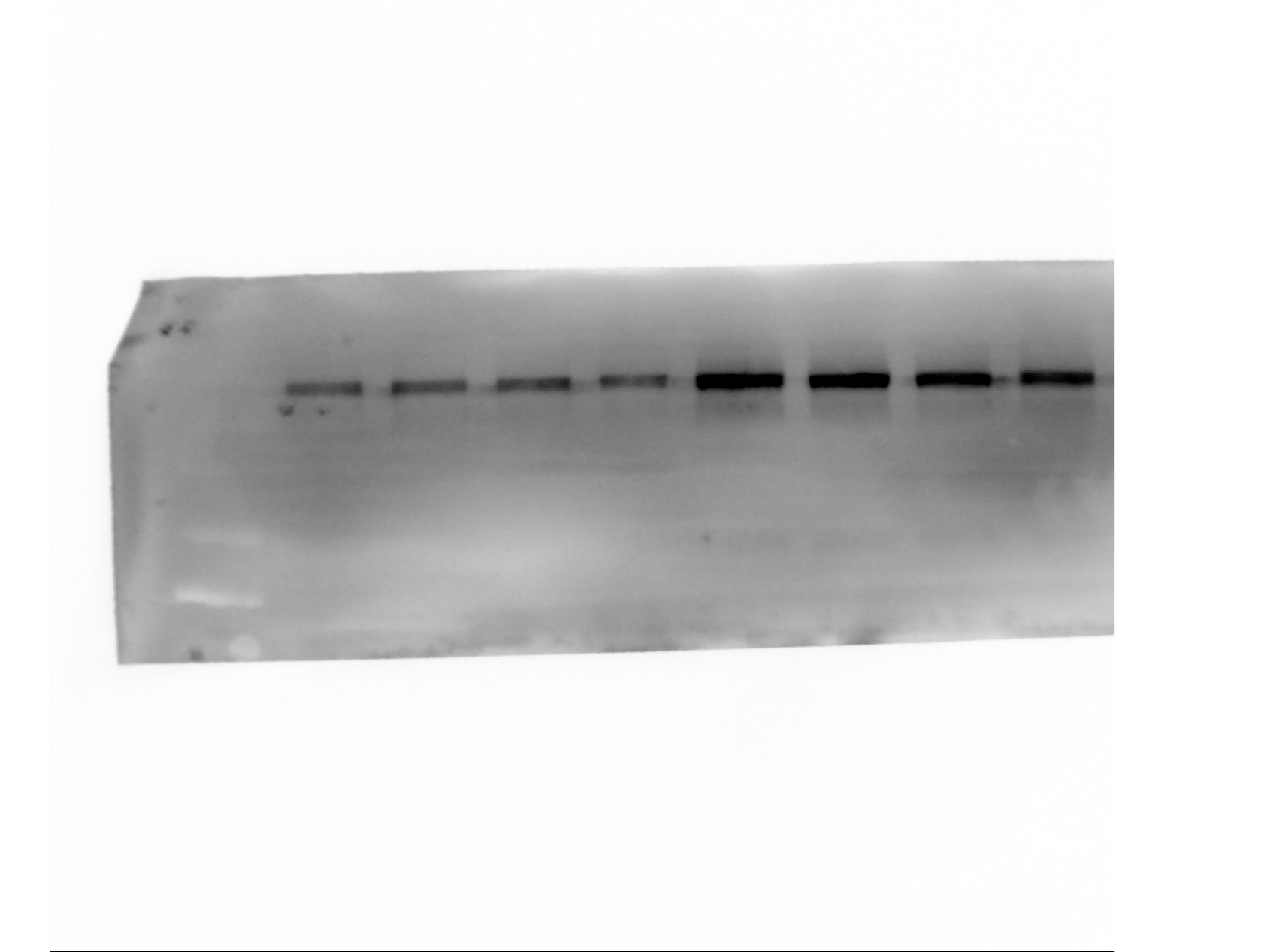

Supplement: S3 Fig — (TIF) [file pone.0309598.s003.TIF]

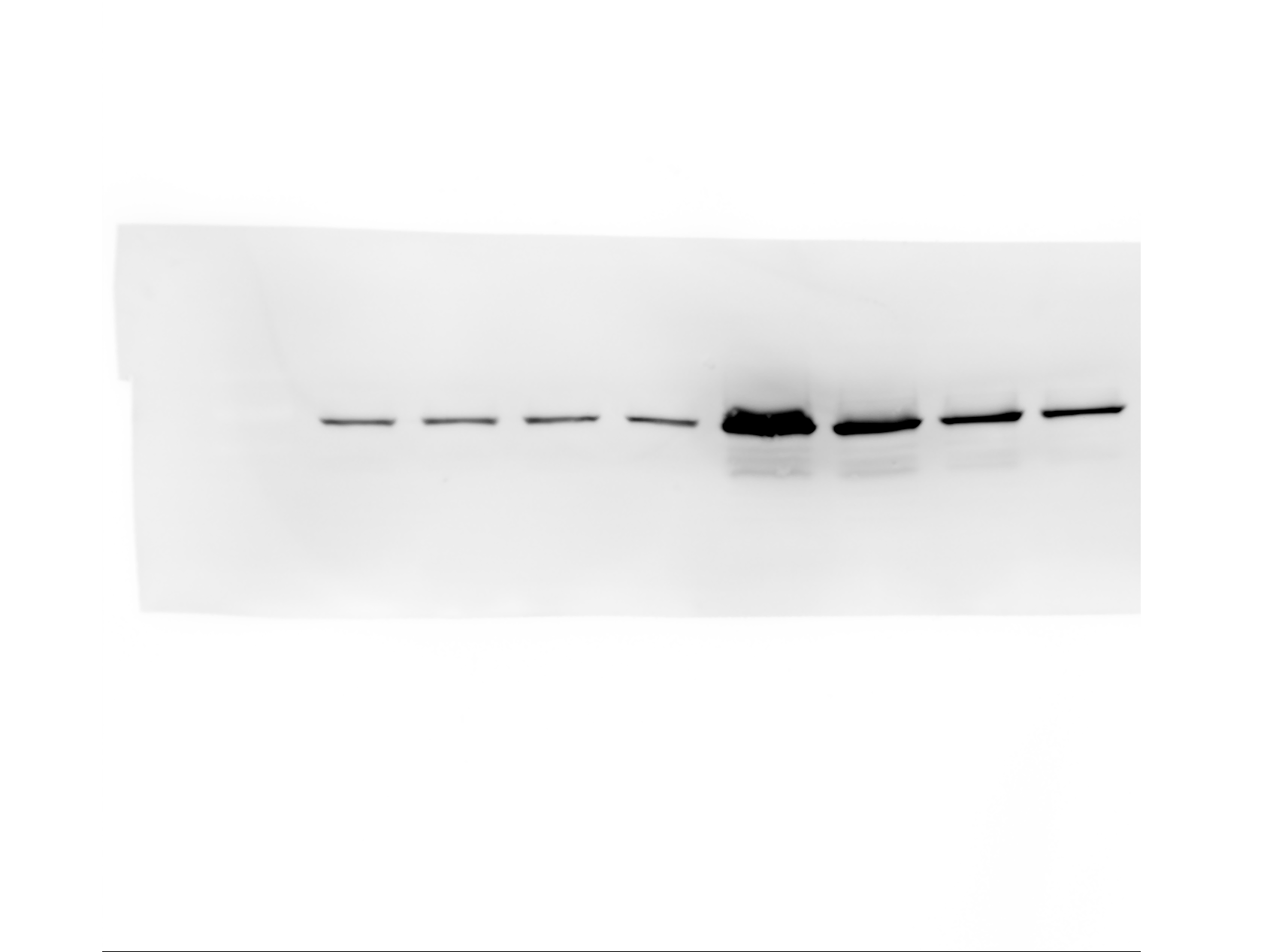

Supplement: S4 Fig — (TIF) [file pone.0309598.s004.TIF]

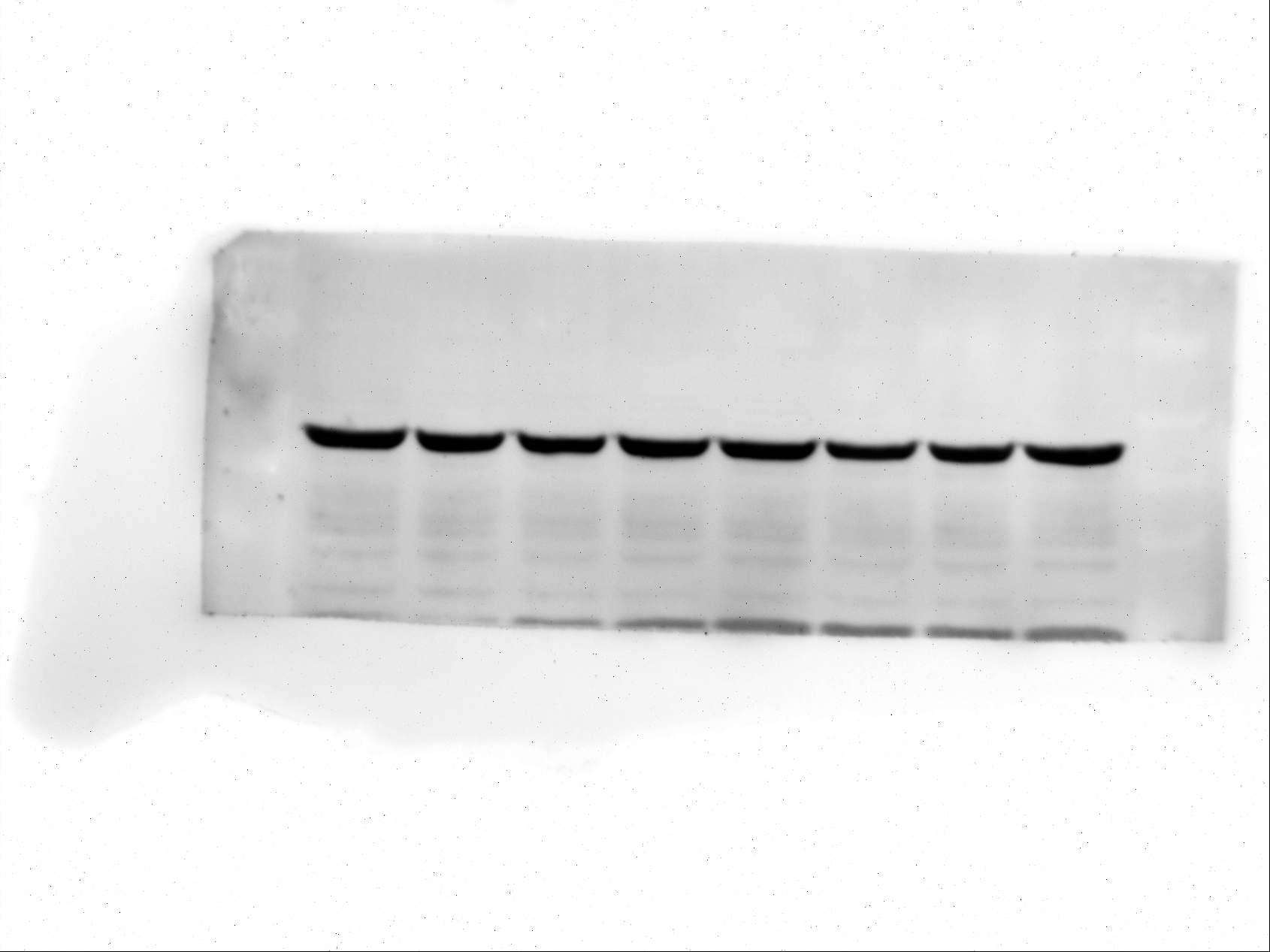

Supplement: S5 Fig — (TIF) [file pone.0309598.s005.TIF]
